# Intelligent Spectrum Sensing for NOMA Systems: A Cost-Sensitive LightGBM Approach with Objective-Driven Learning

**DOI:** 10.3390/s26061767

**Published:** 2026-03-11

**Authors:** Kanabadee Srisomboon, Luepol Pipanmekaporn, Akara Prayote, Wilaiporn Lee

**Affiliations:** 1 Communication and Computer Network Research Group (C2NRG), Electrical Engineering, Department of Electrical and Computer Engineering, Faculty of Engineering, King Mongkut’s University of Technology North Bangkok, Bangkok 10800, Thailand; kanabadee.s@eng.kmutnb.ac.th; 2 Department of Computer and Information Science, Faculty of Applied Science, King Mongkut’s University of Technology North Bangkok, Bangkok 10800, Thailand; luepol.p@sci.kmutnb.ac.th (L.P.); akara.p@sci.kmutnb.ac.th (A.P.)

**Keywords:** non-orthogonal multiple access (NOMA), spectrum sensing, cognitive radio networks, LightGBM, cost-sensitive learning, SPOTIS method, eigenvalue-based features

## Abstract

In NOMA-enabled CR systems, superposed PU signals with unequal power levels and independent activity significantly complicate spectrum sensing and channel state discrimination. To address this issue, ML-based sensing exploits spectrum-domain features to perform channel state classification. However, the ML-based methods remain limited under independent PU activity and suffer from the performance tradeoff issue since the spectrum sensing constraints are not explicitly incorporated into the learning process. In this paper, we propose an OCL method that aligns LightGBM multiclass training with spectrum sensing objectives and leverages eigenvalue-based features to capture discriminative signal patterns under dynamic NOMA transmission. The cost-sensitive learning strategy is used to guide the classifier while the objective-driven tuning is used to optimize hyperparameters toward spectrum sensing objectives. To evaluate the overall performance toward $P_{d}$ and $P_{fa}$, we propose an overall sensing ability score by adopting the SPOTIS method. As a result, the proposed OCL method achieves the highest overall sensing ability scores with an average score of 0.638, outperforming EBSS-RF at 0.610 and FBSS-LR at 0.221. Under challenging signal pattern discrimination conditions, the OCL method improves the overall sensing ability score by 6.26% and 0.9 under different power coefficients compared to EBSS-RF, highlighting its effectiveness in addressing the performance tradeoff issue.

## 1. Introduction

Along with the advancement of fifth-generation (5G) and sixth-generation (6G) networks, the accelerated expansion of smart wireless devices has resulted in severe spectrum congestion and scarcity [1]. Meanwhile, conventional static spectrum allocation [2,3,4] is limited in efficiency and scalability for wireless communication systems since it assigns fixed frequency bands to users. Therefore, licensed bands remain severely underutilized due to the intermittent usage behavior of licensed/primary users (PUs), while unlicensed/secondary users (SUs) are prohibited from accessing them, even when they experience excessive congestion. Currently, several promising technologies have been proposed; however, no single solution satisfies future wireless system requirements, thereby encouraging integrated approaches.

The integration of cognitive radio (CR) [5] and nonorthogonal multiple access (NOMA) [6] presents a promising solution to improve spectrum efficiency and support massive user connectivity in emerging smart wireless networks over limited bandwidth. NOMA allows multiple users to share the same time–frequency resources via power-domain multiplexing with different [7] power levels assigned according to channel conditions, while CR enables the SUs to utilize the licensed bands while avoiding PU reactivation and harmful interference. The integration of NOMA into CR networks is one of the promising solutions [8] that allows the SU to access the licensed band together with PUs when the number of active PUs does not fully occupy the NOMA channel capacity [9]. To satisfy the interference constraint, spectrum sensing and channel estimation are employed to acquire information about the interference channels toward PUs [10,11], thereby enabling SUs to adaptively adjust their transmission parameters to meet the imposed interference constraints. Thus, SUs must not only detect the idle or busy state of a PU channel but also identify the number of active PUs.

In downlink NOMA [12], the base station (BS) transmits superimposed signals of multiple users over the same subcarrier with different power levels while the successive interference cancellation (SIC) method is exploited at the receiver to decode the signal. However, conventional spectrum sensing techniques in CR, such as energy detection [13] and maximum eigenvalue detection, cannot provide reliable discrimination of PU activity in NOMA-enabled CR systems due to signal superposition. In contrast, feature-based spectrum sensing (FBSS) [14,15] has emerged as an alternative by exploiting discriminative signal features; however, its performance is limited under low signal-to-noise ratios (SNRs) of NOMA environments. Moreover, the detection task becomes more challenging under varying PU activity patterns, such as scenarios where only a single PU occupies the licensed channel, as well as under low SNR conditions resulting from path loss.

To overcome the limitations of conventional CR spectrum sensing, machine learning (ML) has been employed to enhance spectrum sensing performance without relying on predefined detection thresholds. In [16], the energy detection is combined with logistic regression, k-nearest neighbor (KNN), and neural networks to investigate the effectiveness of classification models. The simulation results indicate that the effectiveness of classification models enhances the spectrum sensing performance as compared to conventional energy detection.

In [17], the NOMA spectrum sensing problem is formulated as a multi-hypothesis problem where the particle swarm optimization is adopted with a Gaussian mixture model (GMM) to estimate channel coefficients. In [18], deep learning models, such as convolutional neural networks (CNNs) and recurrent neural networks (RNNs), are adopted in spectrum sensing. To extract features from the sequential data of signal, a one-dimensional convolutional neural network (1DCNN) was employed to determine the signal pattern of the PU and determine the channel state using a time series classification model such as a long short-term memory network [19]. Deep learning models have a strong capability to extract features, allowing them to achieve superior detection accuracy without relying on predefined detection thresholds. Nevertheless, the non-cooperative spectrum sensing of NOMA-based spectrum sensing suffers from the performance tradeoff issue [20] in which the spectrum sensing only maximizes the probability of detection ($P_{d}$) while failing to minimize the probability of false alarm ($P_{fa}$). Thus, the cooperative NOMA-based spectrum sensing is proposed to address the performance tradeoff issue. In [21], the ML-driven method is also exploited in cooperative NOMA-based spectrum sensing to address the performance tradeoff issue by applying both unsupervised methods—K-means clustering and Gaussian mixture models—and supervised models—support vector machine (SVM), KNN, and neural networks—to detect multi-PU activity. Even if the cooperative spectrum sensing can address the performance tradeoff issue, it comes at the cost of computational complexity. Therefore, we focus on the non-cooperative spectrum sensing method.

According to multiuser discrimination ability, the spectrum sensing ability of FBSS is enhanced by employing the classification ability of ML [22], where the channel states can be determined by 0.5 of the threshold. By incorporating logistic regression (LR) with FBSS features, the NOMA-based spectrum sensing performance is improved via varying SNR conditions and allocated power coefficients. Eigenvalue-based channel state classification (EBSS) with ML is introduced in [23], where dominant eigenvalues extracted via eigen-decomposition are used as features. Evaluations with LR, Naive Bayes (NB), and Random Forest (RF) under NOMA-based spectrum sensing show that RF performs best, and EBSS outperforms energy-based and maximum-eigenvalue methods in accuracy and F1-score.

### Motivation and Contribution

While integrating NOMA into cognitive radio networks, conventional CR-based spectrum sensing techniques become inadequate for reliable channel state detection, as they are typically designed under binary hypothesis assumptions. Under the superposition of unequal-power PU signals and time-varying PU activity in low-SNR environments, overlapping signal patterns severely hinder channel state discrimination, posing a critical challenge for NOMA-based spectrum sensing. In this paper, we proposed an objective-driven cost-sensitive learning for Light Gradient Boosting Machine-based spectrum sensing (OCL) method under dynamic PU activity in a NOMA downlink scenario where the contribution is three-fold.

First, we introduce a cost-sensitive learning strategy integrated with the Light Gradient Boosting Machine (LightGBM) [24,25] to construct a probabilistic classifier under NOMA-based spectrum sensing with dynamic PU activity. To reflect this practical scenario, we reformulate the binary classification problem of conventional spectrum hypothesis into multiclass hypotheses to infer idle channel, weak PU, strong PU, and superposed PU activity. Using the cost-sensitive learning strategy [26], the multiclass loss regulates the relative importance of PU-active classes and leads the training behavior toward spectrum sensing objectives. Therefore, the proposed OCL method improves the baseline LightGBM training behavior and can address the performance tradeoff issue of non-cooperative NOMA-based spectrum sensing.

Second, we introduce the objective-driven tuning framework that optimizes the LightGBM hyperparameters and class weights under spectrum sensing constraints. Once the baseline selects hyperparameters based on a single global metric, such as overall accuracy, class weights are either predefined based on dataset imbalance ratios or tuned independently from the structural hyperparameters. This disjointed strategy is ill-suited for cognitive radio networks, where spectrum sensing inherently involves the dual and often conflicting objectives of maximizing $P_{d}$ while rigorously constraining the probability of false alarm $P_{fa}$. Consequently, conventional tuning often results in a suboptimal sensing tradeoff, where high detection is achieved at the cost of excessive false alarms. The proposed objective-driven tuning avoids this inconsistency by jointly optimizing the LightGBM hyperparameters and cost-sensitive weights under explicit spectrum sensing constraints. This joint optimization ensures that the decision boundaries are explicitly calibrated to align with the desired $P_{d}$ and $P_{fa}$ targets. As a result, the proposed method maintains strong overall sensing performance across transmission distances.

Third, we introduce the alternative evaluation metric—overall sensing ability— by adopting the multicriteria ranking method—stable preference ordering toward ideal solution (SPOTIS) [27]—to determine an integrated score of the probability of detection ($P_{d}$) while controlling the probability of false alarm ($P_{fa}$). Once the ideal sensing target corresponds to the desired maximized $P_{d}$ and minimized $P_{fa}$, the overall sensing score enables a fair and unified assessment of sensing methods. The results demonstrate that the proposed OCL method provides the most reliable overall performance compared with the baselines considered.

The remainder of this paper is organized as follows: Section 2 describes the concept of NOMA-based spectrum sensing and the problem statement. The proposed OCL method, including its mathematical formulations, is explained in detail in Section 3. In Section 4, the effectiveness of the proposed OCL is compared against baseline LightGBM under classification metrics, and then it is compared to the existing NOMA-based spectrum sensing methods. Finally, Section 5 presents the conclusion.

## 2. NOMA-Based Spectrum Sensing

In this section, we describe the system model and the issue of PU activity on NOMA-based spectrum sensing. In the NOMA downlink system, the BS of the licensed network, or primary network (PRN), performs power-domain superposition according to the channel quality of each PU, resulting in a composite received waveform at the SU receiver. To enable NOMA spectrum sensing with active PU number identification, the sensing problem must be formulated as a multiple-hypothesis problem accounting for the absence or the presence of one or more PUs.

### 2.1. System Model

Consider that k PUs are simultaneously served on the same time–frequency resource, with different power levels assigned by the NOMA-PRN base station. The transmitted NOMA signal is constructed by the power-domain superposition, where the baseband signals of multiple PUs are combined with a different power allocation coefficient ($\alpha_{i}$) according to their channel conditions. Then, the composite NOMA signal (s) can be formulated as

(1) $$s\left(n\right)=\sum_{i=1}^{k}\sqrt{\alpha_{i}\sigma_{s}^{2}}u_{i}\left(n\right),$$

where PUs experiencing poorer channel conditions are assigned lower $\alpha_{i}$ with $\alpha_{i}\;>\;0$, $u_{i}\left(n\right)$ is the signal of *i*th PU, and $\sigma_{s}^{2}$ denotes the average symbol power of *i*th PU.

From the perspective of NOMA-based spectrum sensing, the SU focuses on identifying channel occupancy and the level of PU activity rather than decoding individual PU signals. Depending on the number of active PUs sharing the NOMA channel, the received signal can be modeled under a set of composite hypotheses. Therefore, the hypothesis ($H_{c}$) represents the channel state determined by the active PUs, where *c* ∈ {1, 2, …, *k*}, while $H_{0}$ denotes an empty channel where all PUs are inactive. Then, the signal received ($r\left(n\right)$) at the SU can be written as

(2) $$r\left(n\right)=\left\{\begin{array}{cc} \;\;w\left(n\right) & {,H}_{0}\\ \sum_{i=1}^{k}\sqrt{\alpha_{i}\sigma_{s}^{2}}\;PL\left(d\right)\;u_{i}\left(n\right)+w\left(n\right) & ,H_{c} \end{array}\right.,$$

and

(3) $$PL\left(d\right)=PL\left(d_{0}\right){\left(\frac{d}{d_{0}}\right)}^{\eta },$$

where $PL\left(d\right)$ denotes the large-scale path loss in the linear scale at distance $\left(d\right)$ between the NOMA-PRN base station and the SU, $d_{0}$ is reference distance, η is the path-loss exponent, and $w\left(n\right)$ is circularly symmetric complex additive white Gaussian noise (AWGN).

### 2.2. Spectrum Sensing Techniques for NOMA System

To perform channel state classification, ML models were employed using signal features traditionally considered in conventional spectrum sensing techniques. Among these, eigenvalue-based features and cyclostationary features are two of the most widely used representations for training ML-based classifiers. The cyclostationary features can be determined by the correlation between the received signal and its delay, while the eigenvalues can be obtained using the eigen-decomposition theorem. The cyclostationary features can be expressed by

(4) $$F\left(\varphi \right)=\frac{1}{N_{s}}\sum_{n=0}^{N_{s}-1}r\left(n\right)r*\left(n+\varphi \right),$$

where $N_{s}$ is the length of the received signal sample and φ is the sample shift. Once we employ the eigenvalues as the input features of the proposed method, the details of the eigenvalues calculation will be explained in the next section.

Currently, several machine learning (ML) models have been adopted for ML-based spectrum sensing, where logistic regression and random forest have demonstrated strong performance, reported in [21,23]. In addition, a deep learning model, one-dimensional convolutional neural network (1DCNN) [19], is employed for spectrum sensing due to its capability to effectively extract and classify one-dimensional signal features. Therefore, these models are described in detail in this section.

#### 2.2.1. Logistic Regression

Logistic regression [15] serves as a statistical tool for binary classification, predicting class labels from observed features by transforming a linear combination of inputs into a probability between 0 and 1 using the sigmoid function. By employing the sigmoid function, the final sensing decision of logistic regression (${\hat{y}}_{LR}$) can determined by positive class probability and expressed by

(5) $${\hat{y}}_{LR}=\frac{1}{1+e-(\theta_{0}+\theta_{1}\beta_{1}+\theta_{2}\beta_{2}+\cdots +\theta_{p}\beta_{m})},$$

where $\beta_{i}$ is the *i*th feature, $\theta_{0}$ is the intercept, $\theta_{ith}$ is the coefficients, and m is the number of features.

#### 2.2.2. Random Forest

Random forest is an ensemble technique that builds multiple decision trees and combines their outputs to boost accuracy while curbing overfitting for better generalization. Each tree trains on a bootstrapped sample of the data (drawn with replacement), using a random subset of features for split decisions at every node—this promotes tree diversity and cuts down correlation between them.

For classification, the final prediction for input β can be obtained by majority vote, which is given by

(6) $${\hat{y}}_{RF}=mode\left\{c_{1}\left(\beta \right),c_{2}\left(\beta \right),\ldots ,c_{B_{Tree}}\left(\beta \right)\right\},$$

where $c_{i}\left(\beta \right)$ is the *i*th tree’s output and $B_{Tree}$ is the total number of trees; regression uses averaging instead.

Random forest excels with noisy data, shows low sensitivity to hyperparameters, and handles nonlinear feature relationships effectively. It also offers feature-important scores for insights, despite the ensemble’s black-box nature, making it ideal for high-dimensional, mixed-type datasets across many tasks.

#### 2.2.3. 1DCNN

The 1D Convolutional Neural Network (1D-CNN) is a deep learning architecture designed to extract discriminative features from sequential data such as in-phase (I) and quadrature-phase (Q) radio signals. The objective of the 1DCNN model is to learn a nonlinear decision function, which is given by

(7) $${\hat{y}}_{1DCNN}=f\left\{X_{I},\;X_{Q}\right\},$$

where $X_{I}$ and $X_{Q}$ represent the in-phase and quadrature components of the received signal.

In a 1D-CNN, convolution is performed along the time dimension of the input sequence. For an input vector r, the output of the l-th convolutional layer is expressed as

(8) $$\nu_{ij}^{\left(l\right)}=\sigma \left(b_{j}^{\left(l\right)}+\sum_{h=1}^{K}w_{h,j}^{\left(l\right)}r_{i+h-1,j}^{\left(l-1\right)}\right),$$

where K is the kernel size, $b_{j}^{\left(l\right)}$ is the bias, $w_{h,j}^{\left(l\right)}$ are convolution kernel weights, and $\sigma \left(\cdot \right)$ is the activation function.

The convolutional layers act as automatic feature extractors, capturing local temporal correlations and energy patterns in the received signal. After convolution and flattening, the learned feature vector is forwarded to fully connected (FC) layers, which perform the final classification.

### 2.3. Problem Statement

Due to the power-domain superposition of multiple PUs in the same channel, the statistical characteristics of the received signal vector (r) significantly distort based on unequal power levels of multiple PU signals. Therefore, it highlights the fundamental challenge of spectrum sensing in NOMA-enabled cognitive radio networks, where conventional methods such as energy detection and correlation-based sensing experience severe performance degradation, particularly under low-SNR conditions and strong PU power imbalance.

To overcome these limitations, ML-based spectrum sensing approaches have been adopted to model complex signal characteristics. However, existing ML-based solutions still suffer from two fundamental limitations. First, commonly used features fail to adequately represent channel occupancy and the PU activity in NOMA systems, which further degrades sensing reliability. Second, the objective functions are often misaligned with spectrum sensing objectives that aim to maximize detection probability under constrained false alarm rates.

## 3. Objective-Driven Cost-Sensitive Learning for LightGBM-Based Spectrum Sensing

In this section, we propose an objective-driven cost-sensitive learning strategy, referred to as objective-driven cost-sensitive learning for LightGBM-based spectrum sensing (OCL), to align LightGBM multiclass classification with the composite hypotheses of NOMA spectrum sensing. In our proposed method, eigenvalue-based features are used to capture the statistical characteristics of r. The cost-sensitive learning strategy [26] is adopted to regulate the training behavior toward spectrum sensing objectives by formulating the heterogeneous PU activity states into multiclass a classification problem. Moreover, the objective-driven tuning is used to optimize both the LightGBM [24,25] hyperparameters and class weights under spectrum sensing constraints.

**Figure 1:**
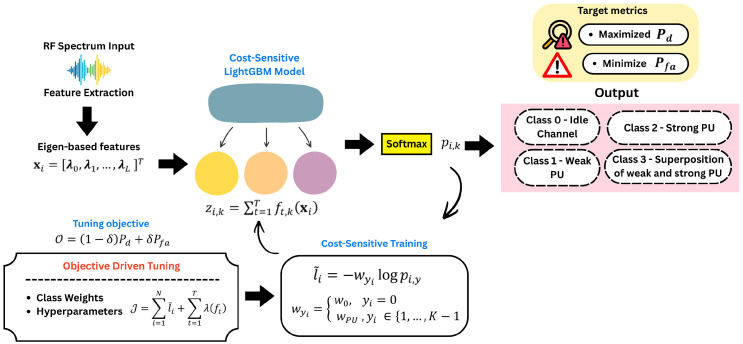
Objective-driven cost-sensitive learning framework.

As shown in Figure 1, the workflow of the OCL method is illustrated. After the signal is received, it is transformed into an eigenvalue-based representation that captures the underlying second-order statistical structure of the signal. These extracted features are then fed into a cost-sensitive LightGBM classifier, which generates class-wise raw scores through an additive ensemble of leaf-wise regression trees, and converts them into posterior class probabilities via a softmax mapping. To obtain the best model, the objective-driven tuning loop is employed to jointly optimize the class weights and LightGBM hyperparameters under spectrum sensing constraints, aiming to maximize $P_{d}$ performance while controlling $P_{fa}$. Finally, the optimized probabilistic outputs are used to infer the multiclass spectrum occupancy state, enabling reliable discrimination among idle channels, weak PU, strong PU, and superposed PU activity.

### 3.1. Eigen-Based Feature Extraction

Because r consists of a superposition of multiple PU signals with unequal power levels, a particular signal feature, such as energy, does not uniquely characterize the underlying spectrum state. In our proposed method, the eigenvalue-based features are adopted to capture the statistical structure of the received signal under different channel occupancy conditions.

Let $r_{i}\in C^{N_{s}}$ represent the complex baseband received signal vector within the ith window. Then, the sample covariance (R) can be estimated as

(9) $$R=E\left[r_{i}{r_{i}}^{H}\right],$$

where ${\left(\cdot \right)}^{H}$ is the Hermitian transpose.

Then, the eigenvalues corresponding to the eigenvectors are calculated by eigendecomposition, which is expressed by

(10) $$R=Q\Lambda Q^{H},$$

where Q is the unitary matrix of eigenvectors, $\Lambda =diag\left(\lambda_{0},\;\lambda_{1},\ldots ,\lambda_{L}\right)$ is the diagonal matrix of eigenvalues, and L is number of samples of observation vector.

In our proposed method, we obtain the eigenvalues as the input features of the LightGBM classifier, which determines the channel occupancy condition since it represents the second-order statistical structure of the received signal. Therefore, the eigen-feature vector is constructed as $x_{i}={\left[\lambda_{0},\;\lambda_{1},\ldots ,\lambda_{L}\;\right]}^{T}$. Although eigenvalue-based features come at a high computational cost, we mitigate this issue by employing a fixed snapshot length L. Therefore, the eigenvalue extraction is performed on a small Hermitian covariance matrix, leading to a linear-time complexity with respect to the signal length per sensing window.

### 3.2. LightGBM-Based Spectrum Sensing Framework

To determine the channel occupancy condition, LightGBM is adopted with a cost-sensitive learning strategy, where the classification model is trained using class-dependent weighting in the multiclass cross-entropy loss. Therefore, the LightGBM classifier reflects asymmetric misclassification costs among noise and PU activity. In this work, we formulate the multiclass NOMA spectrum sensing problem as a probabilistic classification problem with four channel states, including noise-only, weak PU, strong PU, and superposition of weak and strong PUs. Then, the corresponding spectrum class label can be denoted as $y_{i}\in \left\{0,1,\ldots ,K-1\right\}$ where K is number of channel states.

By employing LightGBM as the channel condition classifier, the multiclass sensing output is obtained as a class-wise raw score vector formed by aggregating the contributions of multiple regression trees in an additive ensemble manner. It should be mentioned that the class-wise raw score vector is used instead of hard decisions to retain confidence information across multiple PU activity hypotheses. Then, the class-wise raw score ($z_{i,k}$) for class k is constructed as

(11) $$z_{i,k}=\sum_{t=1}^{T}f_{t,k}\left(x_{i}\right),\;\;\;\;\;\;k\in \left\{0,1,2,3\right\},$$

where $f_{t,k}\left(\cdot \right)$ is the tth regression tree associated with class k and T is the number of boosting trees.

It can be mentioned that the class-wise raw score is formed by summing the outputs of leaf-wise regression trees, where each tree partitions the eigen-feature space into disjoint regions ${\left\{R_{t,l}\right\}}_{l=1}^{L_{t}}$ and assigns a constant value per region. A piecewise-constant leaf can be expressed as

(12) $$f_{t,k}\left(x_{i}\right)=\sum_{l=1}^{L_{t}}v_{t,k,l}I\left(x_{i}\in R_{t,l}\right),$$

where $L_{t}$ is the number of leaves at iteration, $v_{t,k,l}$ is the leaf value for class k, and $I\left(\cdot \right)$ is the indicator function, which equals one if the condition is satisfied and otherwise is zero.

Since $z_{i,k}$ represents a class-wise raw score rather than a probability, the posterior probabilities ($p_{i,k}$) are computed by applying the softmax function to the class-wise raw scores as

(13) $$p_{i,k}=P\left(y_{i}=k|x_{i}\right)=\frac{e^{z_{i,k}}}{\sum_{j=1}^{K}e^{z_{i,j}}}.$$

Then, the final sensing decision (${\hat{y}}_{i}$) is formulated by

(14) $${\hat{y}}_{i}=\arg\underset{k}{\max}\left(p_{i,k}\right).$$

### 3.3. Cost-Sensitive Learning Toward Spectrum Sensing

Once the baseline LightGBM assigns equal cost to all misclassifications, its optimization strategy is not explicitly aligned with spectrum sensing requirements, which emphasize maximizing the probability of detection ($P_{d}$) while controlling the probability of false alarm ($P_{fa}$). In cognitive radio spectrum sensing, missed detection is more harmful from the spectrum sensing perspective than a false alarm, and this is exacerbated as high-power PUs dominate the received signal and mask low-power PU activity under NOMA transmission. Then, we adopt a cost-sensitive learning strategy based on weighted multiclass cross-entropy to bias learning toward spectrum sensing performance.

By assigning class-dependent cost-sensitive weight to the loss function of baseline LightGBM, the weighted cross-entropy loss (${\tilde{l}}_{i}$) is formulated by

(15) $${\tilde{l}}_{i}=-w_{y_{i}}\log p_{i,y_{i}},$$

where $w_{y_{i}}$ is the class-dependent weight associated with the true spectrum state $y_{i}$ and

(16) $$w_{y_{i}}=\left\{\begin{array}{c} \;w_{0},\;y_{i}=0\;\;\;\;\;\;\;\;\;\;\;\;\;\;\;\;\;\;\;\;\;\;\;\;\;\;\\ w_{PU}\;,\;y_{i}\;\in \left\{1,\ldots ,K-1\right\} \end{array}\right..$$

Then, the weight $w_{y_{i}}$ is used to elicit control over the tradeoff between missed detection and false alarm behavior in spectrum sensing. The overall objective function (J) for LightGBM training is then given by

(17) $$J=\sum_{i=1}^{N}{\tilde{l}}_{i}+\sum_{t=1}^{T}\lambda \left(f_{t}\right),$$

where $\lambda \left(f_{t}\right)$ is the regularization term controlling tree complexity.

To enable second-order boosting in LightGBM for the considered multiclass spectrum sensing problem, the first-order (gradient) and second-order derivatives (Hessian matrix) of the cost-sensitive loss with respect to the class-wise raw score $z_{i,k}$ for each class can be determined by

(18) $$g_{i,k}≜\frac{\partial {\tilde{l}}_{i}}{\partial z_{i,k}}=w_{y_{i}}\left(p_{i,k}-y_{i,k}\right),k\in \left\{0,1,2,3\right\},$$

and

(19) $$h_{i,k}≜\frac{\partial^{2}{\tilde{l}}_{i}}{\partial z_{i,k}^{2}}=w_{y_{i}}p_{i,k}\left(1-p_{i,k}\right),k\in \left\{0,1,2,3\right\},$$

where $g_{i,k}$ is the gradient term and $h_{i,k}$ is the diagonal Hessian term. Then, the cost-sensitive learning directly rescales the gradient and second-order statistics in the LightGBM optimization process by regulating the model bias toward PU-active classes to better align with spectrum sensing objectives.

### 3.4. Objective-Driven Tuning Under Spectrum Sensing Constraints

While cost-sensitive weighting regulates the learning behavior, the optimal balance between $P_{d}$ and $P_{fa}$ can be achieved by tuning the class weights and model hyperparameters. Therefore, an objective-driven tuning framework is introduced to optimize both hyperparameters and class weights under spectrum sensing criteria.

By comparing classification to spectrum sensing metrics, $P_{d}$ can be determined by the correct classification of PU active states $k\in \left\{1,2,3\right\}$, and $P_{fa}$ is misclassification of class k=0. Then, $P_{d}$ and $P_{fa}$ can be determined by classification metrics as

(20) $$P_{d}=\frac{\left({Recall}_{1}+{Recall}_{2}+{Recall}_{3}\right)}{3},$$

and

(21) $$P_{fa}=1-{Recall}_{0},$$

where ${Recall}_{k}$ is the recall of class k.

Then, the tuning objective (O) is formulated as

(22) $$O=\left(1-\delta \right)P_{d}+\delta P_{fa},$$

where δ is the tradeoff weight controlling influence of maximizing detection probability over suppressing false alarms and $\delta \in \left[0,1\right]$.

## 4. Results and Discussion

In this section, we evaluate the NOMA-based spectrum sensing performance of the proposed OCL method in comparison with two existing ML-based spectrum sensing approaches—feature-based spectrum sensing (FBSS) [14,15] and eigen-based spectrum sensing (EBSS) [23]. Before conducting the spectrum sensing comparison with FBSS and EBSS, we first assess the effectiveness of OCL against an eigen-based feature with baseline LightGBM under classification metrics. Subsequently, the spectrum sensing performance of OCL is compared with FBSS and EBSS by varying path loss effects corresponding to PU–SU distances ranging from 10 to 150 m.

### 4.1. Classification-Based Evaluation and NOMA-Based Spectrum Sensing Evaluation Dataset Generation

In our simulations, we generate two independent datasets to separately support model training with classification-based evaluation and spectrum sensing evaluation under realistic channel variations. A synthetic PU signal is generated using QPSK modulation, and the received signal is formed under four channel states: (i) idle channel (noise-only), (ii) weak PU, (iii) strong PU, and (iv) superposition of weak and strong PU. The sample signal length ($N_{s}$) is 4096 and tradeoff weight controlling (δ) is set to 0.5. Under an urban scenario, the path-loss exponent (η) is set to 3.0 while the reference distance ($d_{0}$) is set to 100 m.

To train and validate the multiclass spectrum classifiers, the dataset is constructed to represent four spectrum occupancy states under varying distances. The number of samples per class is explicitly controlled to match the class support used in the classification-based evaluation, i.e., 12,320 samples for Class 0, and 13,200 samples for each PU-active class (Classes 1–3). It should be noted that classes 0, 1, 2, and 3 indicate the idle channel, weak PU, strong PU, and superposition of weak and strong PU, respectively. A fivefold cross-validation protocol is then applied to provide an unbiased assessment under standard classification metrics, while mitigating dependence on a single train–test split and reducing the risk of overfitting or underfitting.

To evaluate NOMA-based sensing performance, we generate a separate spectrum sensing evaluation dataset using distance-oriented Monte Carlo simulations. Specifically, the PU–SU distance is varied across the considered range to emulate different path loss conditions. For each distance, we conduct 5000 Monte Carlo iterations where each iteration generates an independent channel and noise realization together with the corresponding received signal under the four sensing states. All methods are applied to infer the spectrum class, and the spectrum performance metrics—such as the probability of detection and probability of false alarm—are computed and averaged over the 5000 trials. Therefore, the classification performance can be assessed under spectrum sensing constraints.

### 4.2. Classification-Based Performance of OCL Against Baseline LightGBM

First, we compare the classification performance of the proposed OCL method against the baseline LightGBM classifier under five standard metrics. Precision, recall, and F1-score are used to evaluate the classification performance of each class, while the overall accuracy and F1-score are reported in terms of macro-averaged and weighted-average scores. The macro average reflects the mean performance across all classes, whereas the weighted average determines the score by weighting each class according to its number of ground-truth samples.

As shown in Table 1, the classification performance of the baseline LightGBM and OCL are compared when $\alpha_{1}=0.2$ and $\alpha_{2}=0.8$ and when $\alpha_{1}=0.4$ and $\alpha_{2}=0.6$. When $\alpha_{1}=0.2$ and $\alpha_{2}=0.8$, by using eigenvalue-based features, a baseline LightGBM classifier presents high detection performance of the idle channel (Class 0), with a high recall of 0.9167 and moderate precision of 0.6691. When only the weak PU (Class 1) is active, the baseline LightGBM classifier shows moderate and relatively balanced performance, with a precision of 0.7437 and a recall of 0.6248. The most severe performance degradation is observed when only the strong PU (Class 2) is active, where the recall drops to 0.3577 and the F1-score reaches only 0.4372. It can be observed that the performance of the baseline LightGBM classifier suffers from similar eigenvalue-based features among noise, the strong PU, and long-distance of weak PU. In contrast, the superposition of PUs (Class 3) exhibits comparatively stable performance, with a recall of 0.7632 and an F1-score of 0.7008. Overall, the baseline LightGBM achieves a macro-averaged F1-score of 0.6477 and a weighted F1-score of 0.6455.

When the power coefficients are $\alpha_{1}=0.4$ and $\alpha_{2}=0.6$, noticeable changes in precision, recall, and F1-score can be observed for both the baseline LightGBM and the proposed OCL method. Under the idle channel, both models exhibit noticeable improvement on the classification performance, where baseline LightGBM increases its recall 0.9331, while the OCL method further enhances recall to 0.9917. When the PU power coefficients become closer, the eigenvalue-based features of PU-active states deviate more clearly from noise, resulting in a higher F1-score of 0.8090 for baseline LightGBM and 0.8156 for the OCL. In contrast, the recognition of single-user states becomes more challenging as the power coefficients approach similar values. Under weak PU activation, the baseline LightGBM exhibits a clear reduction in recall together with a noticeable decline in F1-score. Although the OCL method preserves relatively high precision, its recall also decreases, indicating increased difficulty in correctly identifying weak-PU activity. When the power coefficients are closer, the eigen-structures of Class 1 and Class 2 become more similar, thereby increasing mutual confusion between these two single-user hypotheses. Under strong PU activation, the baseline model achieves moderate improvements in recall while the OCL method maintains high precision indicating more discriminative decision regions. For the superposed state, the OCL method benefits from clearer eigenvalue separation under balanced power allocation, achieving a recall of 0.8574 and an F1-score of 0.6972, outperforming the baseline model. While the model can reasonably separate the four sensing states overall, it remains unreliable in recognizing strong-PU activity. It can be noticed that the baseline LightGBM training objective treats all misclassification errors equally and thus prioritizes overall accuracy rather than detection-critical PU states. Consequently, strong-PU samples are often misclassified into other PU-active hypotheses.

On the other hand, the OCL method outperforms the overall performance of the baseline LightGBM classifier, achieving an accuracy of 0.6872 with a macro F1-score of 0.6700 and a weighted F1-score of 0.6681 when $\alpha_{1}=0.2$ and $\alpha_{2}=0.8$. Under the idle channel state, the OCL method shows a recall of 0.9739, indicating an improvement achieved by penalizing missed PU activity and reducing false idle declarations relative to the baseline LightGBM. When only weak PU is active, both precision and recall improve, reflecting enhanced separability of weak-PU activity. The superposition state also benefits from a higher recall and F1-score, suggesting more robust discrimination of PU-active conditions under NOMA transmission. Under the strong-PU-only state, the OCL method slightly improves the recall of baseline LightGBM, where the recall is 0.3604. When $\alpha_{1}=0.4$ and $\alpha_{2}=0.6$, the baseline LightGBM achieves an overall accuracy of 0.6528 with a macro F1-score of 0.6416 and a weighted F1-score of 0.6387, while the OCL method improves the overall sensing performance, reaching an accuracy of 0.6984 with a macro F1-score of 0.6842 and a weighted F1-score of 0.6820. Overall, the OCL method improves the calibration of PU-active decision regions and prioritizes detection-critical classes, whereas the baseline LightGBM optimizes generic classification accuracy.

### 4.3. Comparison of NOMA-Based Spectrum Sensing Performance

Second, we evaluate the NOMA-based spectrum sensing performance of the proposed OCL method in comparison with feature-based spectrum sensing (FBSS) and eigen-based spectrum sensing (EBSS). As described in [20], conventional ML-based FBSS employs cyclostationary features with logistic regression (FBSS-LR) to perform spectrum sensing under NOMA-based PU signal superposition. To determine the effectiveness of the OCL method employing eigenvalue-based features, we extend the concept of conventional FBSS by incorporating the proposed cost-sensitive and objective-driven learning strategy with cyclostationary features (FBSS-OCL) to demonstrate the suitability of learning-based feature designs for NOMA spectrum sensing. On the other hand, EBSS is realized using eigen-based features with a random forest classifier (EBSS-RF).

In Table 2, the $P_{d}$ of NOMA-based sensing methods under two conditions of power coefficients are evaluated over PU–SU distances from 10 to 150 m. When $\alpha_{1}=0.2$ and $\alpha_{2}=0.8$, OCL, EBSS-RF, and 1DCNN achieve $P_{d}$ = 1 at short distances of 10–20 m, whereas FBSS-LR drops sharply at 20 m, indicating weak robustness under moderate attenuation. FBSS-OCL improves over FBSS-LR at 20 m but deteriorates rapidly beyond 30 m and becomes extremely low after 60 m since it is sensitive to the path loss effect, leading to unstable sensing performance in the NOMA-based transmission. Although the 1DCNN shows slightly higher $P_{d}$ than OCL between 30 and 80 m, the OCL preserves relatively higher and more stable detection behavior compared to the others at longer distances beyond 90 m. When $\alpha_{1}=0.4$ and $\alpha_{2}=0.6$, the degradation becomes more significant in the mid- to long-distance region. Although OCL, 1DCNN, and EBSS-RF achieve perfect detection at 10–20 m, EBSS-RF performance deteriorates more rapidly beyond 50–60 m because the eigenvalue separation between weak and strong PU states decreases, thereby weakening purely statistical eigenvalue-based discrimination. In the distance range of 30–80 m, OCL and 1DCNN maintain superior and more stable performance relative to FBSS baselines, while EBSS-RF exhibits noticeable instability at larger distances. Beyond 100 m, detection probabilities of all methods decrease significantly. It can be mentioned that the OCL method offers strong nonlinear discrimination for eigen-feature patterns based on LightGBM classifier. By using cost-sensitive weighting, the priority of PU-active states is recognized, leading to the avoidance of missed detection.

As shown in Table 3, the $P_{fa}$ of all methods are compared. When $\alpha_{1}=0.2$ and $\alpha_{2}=0.8$, 1DCNN and FBSS-LR exhibit a consistently high false-alarm level across all distances, indicating the LR-based FBSS decision rule tends to over-declare PU activity and lacks effective false-alarm control under NOMA transmission. Notably, 1DCNN suffers from the temporal representations of the received signal since it may shift the noise–PU region toward more aggressive PU declarations. On the other hand, OCL consistently maintains a low and stable $P_{fa}$ in the range of 0.050–0.059, reflecting conservative and well-regularized decision behavior under noise-dominant conditions. When $\alpha_{1}=0.4$ and $\alpha_{2}=0.6$, all methods exhibit improved idle-channel discrimination compared to the configuration of $\alpha_{1}=0.2$ and $\alpha_{2}=0.8$ since the feature distribution of idle channels is more distinguishable when PU activity occurs. EBSS-RF slightly improves and OCL maintains consistently low and stable $P_{fa}$. However, 1DCNN still shows the worst classification performance in terms of false-alarm behavior. Overall, the results indicate that increasing the power coefficient enhances the contrast between noise and PU-active signal structures, thereby facilitating idle-channel detection. Through LightGBM’s additive boosting mechanism, the OCL method progressively stabilizes the noise–PU boundary in alignment with spectrum sensing objectives. FBSS-OCL achieves the lowest $P_{fa}$ among the compared methods, indicating the cost-sensitive objective regulation further suppresses false alarms within the FBSS feature space. EBSS-RF yields moderate $P_{fa}$ around 0.10 to 0.12, implying that eigen-statistical features combined with random forest provide reasonable robustness but at a higher false-alarm floor than OCL.

**Figure 2:**
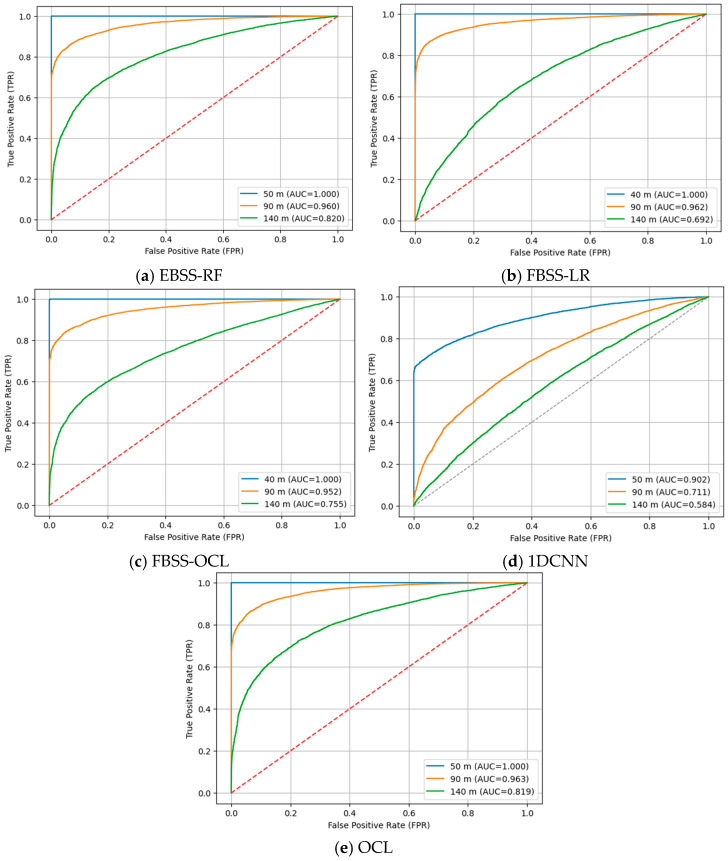
A comparison of ROC curves when $\alpha_{1}=0.2$ and $\alpha_{2}=0.8$.

**Figure 3:**
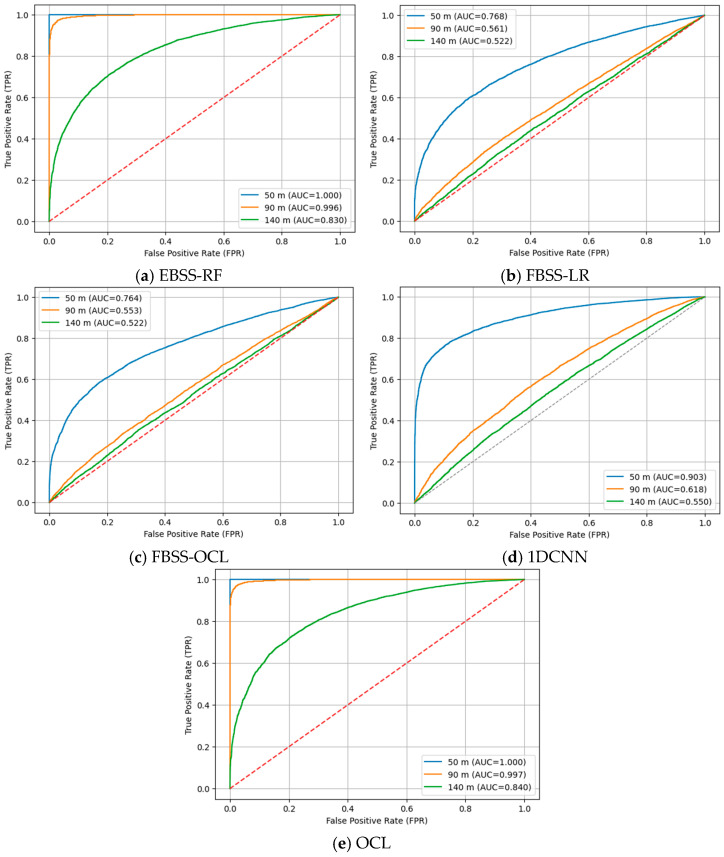
A comparison of ROC curves when $\alpha_{1}=0.4$ and $\alpha_{2}=0.6$.

As depicted in Figure 2, when $\alpha_{1}=0.2$ and $\alpha_{2}=0.8$, all methods show strong separability between idle and PU-active states at 50 m with an AUC of 1.00. As the distance increases to 90 m and 140 m, the classification performance degrades due to path loss, where OCL consistently maintains superior ROC characteristics and achieves the highest AUC values across all evaluated distances, demonstrating more stable and robust discrimination under increasing attenuation. EBSS-RF maintains relatively stable ROC characteristics at a moderate distance with gradual AUC reduction while FBSS-LR and 1DCNN show significant degradation with low values. As depicted in Figure 3, when $\alpha_{1}=0.4$ and $\alpha_{2}=0.6$, all methods still demonstrate strong separability with high AUC values at 50 m. As the distance increases to 90 m and 140 m, OCL consistently maintains the most favorable ROC characteristics and achieves the highest AUC values, while 1DCNN also experiences substantial AUC reduction as distance increases limited capability in learning signal characteristics. EBSS-RF exhibits a more substantial decline in AUC, indicating reduced robustness at extended distances. FBSS-LR shows the most severe AUC degradation while FBSS-OCL achieves moderate AUC improvement over FBSS-LR. Therefore, OCL consistently achieves the highest AUC across all distances, demonstrating the most stable and robust detection performance.

To provide an overall and fair comparison among sensing methods, we adopt the SPOTIS-based ability score as a unified indicator that jointly reflects $P_{d}$ and $P_{fa}$ rather than interpreting them separately. Since the SPOTIS method evaluates sensing quality by measuring the normalized distance from an ideal sensing point where lower scores indicate better overall performance, we define an overall sensing ability score as 1- SPOTIS score to obtain a more intuitive metric in which higher values imply stronger performance. The ideal reference is defined by the desired spectrum sensing targets, i.e., the desired maximum $P_{d}$ is 1, desired minimum $P_{d}$ is 0, desired maximum $P_{fa}$ is 0.4, and desired minimum $P_{fa}$ is 0.

**Figure 4:**
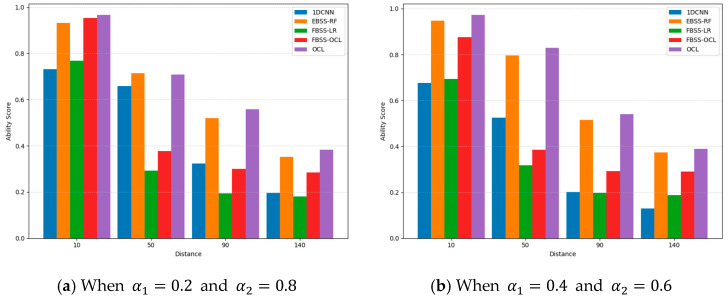
A comparison of overall sensing ability.

As shown in Figure 4, the ability scores at four representative PU–SU distances of 10, 50, 90, and 140 m are illustrated to capture short-, medium-, and long-range sensing conditions under distance-dependent path loss. As the distance increases, all methods exhibit a decreasing trend due to SNR degradation. As shown in Figure 4a, OCL achieves the highest ability score at 10 m, indicating the most favorable sensing tradeoff under strong received signals. As the distance increases, FBSS-LR suffers the most severe performance degradation while FBSS-OCL offers only a slight robustness improvement. EBSS-RF remains relatively competitive at moderate ranges, reflecting the stability of eigen-statistical features with nonlinear learning. On the contrary, the 1DCNN demonstrates moderate performance at 40 m but experiences noticeable degradation as the distance increases, indicating sensitivity to attenuation. By considering all distances, OCL achieves the highest overall ability score of approximately 0.638, while EBSS-RF, FBSS-OCL, and FBSS-LR provide 0.610, 0.385, and 0.221, respectively. As shown in Figure 4b, OCL maintains the strongest overall ability score under $\alpha_{1}=0.4$ and $\alpha_{2}=0.6$, demonstrating consistent robustness across propagation variations. EBSS-RF remains the second-best performer at moderate ranges but shows performance reduction at extended distances. Even if the 1DCNN achieves competitive results at 50 m, its performance significantly degrades at 90 m and 140 m. FBSS-OCL slightly outperforms FBSS-LR, yet both methods remain significantly lower than OCL and EBSS-RF.

Based on the results, when the signal pattern becomes more difficult to discriminate since the distance is over 90 m, the OCL method improves the SPOTIS score by 6.26% compared to EBSS-RF when $\alpha_{1}=0.2$ and $\alpha_{2}=0.8$. Under $\alpha_{1}=0.4$ and $\alpha_{2}=0.6$, EBSS-RF demonstrates improved idle-state discrimination; however, OCL still achieves higher overall ability scores with a 0.9% improvement over EBSS-RF, highlighting its effectiveness in addressing the performance tradeoff issue through the objective-driven cost-sensitive design of OCL. It can be mentioned that insufficient decision boundary of LR leads to the weakest performance among others. Once the OCL maintains the strongest ability score across the considered distances, the proposed method provides more reliable NOMA-based spectrum sensing under propagation variations.

By employing the leaf-wise boosting structure of the LightGBM classifier, the proposed method can effectively distinguish overlapping eigen-feature patterns caused by path loss and PU signal superposition, while cost-sensitive weighting prioritizes PU-active recognition. Moreover, the objective-driven tuning selects the best hyperparameters and weights according to the spectrum sensing constraints. Therefore, the proposed OCL achieves the highest ability scores by offering stronger nonlinear discrimination, whereas logistic regression is constrained by linear separability and random forest focuses on generic impurity reduction without directly controlling detection-oriented behavior. It can be mentioned that the difficulty in discriminating strong-PU states mainly stems from feature overlap under dynamic NOMA transmission. Strong-PU conditions may exhibit eigenvalue and energy patterns similar to superposed or moderate-power states, particularly in the presence of fading and noise variations. Consequently, the decision boundaries become less separable in the feature space, increasing the risk of misclassification.

### 4.4. Computational Complexity Analysis

Finally, we compare the inference-time computational complexity of OCL against FBSS-LR, EBSS-RF, and 1DCNN using Big O notation since the performance of spectrum sensing is primarily constrained by online decision latency. Then, the comparison focuses exclusively on inference rather than offline training.

During inference, FBSS extracts cyclostationary features by processing the received signal in the frequency domain across multiple cyclic frequencies while the LR classifier is used to determine channel state. Once the FBSS feature dimension is small, the overall inference complexity of FBSS-LR can be expressed by

(23) $$O\left(C_{\alpha }N_{s}logN_{s}\right),$$

where $C_{\alpha }$ is the number of cyclic frequencies.

For EBSS-RF, inference consists of two stages including eigenvalue-based feature extraction and RF classification. The eigen-based feature extraction employs a fixed snapshot length and block-wise covariance estimation; consequently, the inference complexity per sensing window scales linearly with the signal length. Meanwhile, the RF classifier performs inference by traversing each decision tree. Therefore, the overall inference complexity of EBSS-RF is dominated by

(24) $$O\left(N_{s}+B_{Tree}D\right),$$

where $B_{Tree}$ is the number of trees and D is the maximum tree depth.

Similar to EBSS-RF, the proposed OCL extracts the received signal features by employing eigenvalue decomposition, while the LightGBM model is used as the classification method. During inference, each tree is traversed from the root to a leaf node requiring a depth-limited sequence of comparisons. Therefore, the overall inference complexity can be expressed as

(25) $$O\left(N_{s}+T\right),$$

where T is the number of decision trees.

For the 1DCNN, inference is composed of two main stages, including convolutional feature extraction over the IQ sequence and classification using the neural network. The inference consists of convolutional feature extraction followed by a global pooling and a linear classification layer. Thus, the inference-time computational complexity per sensing window can be approximated as

(26) $$O\left(KN_{s}C_{0}^{2}2^{B}\right),$$

where $C_{0}$ is the base channel width and B is the number of convolutional blocks.

It can be mentioned that the FBSS-LR approach relies on FFT-based cyclostationary feature extraction, which incurs a higher computational cost during inference. In contrast, both EBSS-RF and the proposed OCL exhibit linear-time feature extraction complexity due to the use of eigenvalue-based features with a fixed snapshot length. While EBSS-RF achieves classification performance through ensemble diversity using a large number of independently trained trees, OCL exploits boosting-based representation efficiency, enabling improved detection performance with fewer model components. For the 1DCNN, inference is entirely driven by convolutional feature extraction over the raw IQ sequence, followed by global pooling and a linear classifier. Unlike the tree-based methods whose complexity grows primarily with signal length and tree depth, the computational cost of 1DCNN is dominated by convolutional operations. Therefore, compared to EBSS-RF and OCL, the 1DCNN generally requires higher inference-time computational resources.

## 5. Conclusions

In this paper, we propose an OCL method for NOMA-enabled cognitive radio systems which is explicitly designed to address spectrum sensing under superposed unequal-power PU signals and independent PU activity. To address the performance tradeoff of existing machine-learning–based sensing approaches, OCL formulates spectrum sensing as a constraint-aware multiclass learning problem, while others rely on conventional binary assumptions without considering spectrum sensing constraints. By jointly optimizing LightGBM hyperparameters and cost-sensitive loss weights, the proposed method directly aligns classifier training with detection objectives while leveraging the strong discriminative capability of eigenvalue-based features. Comprehensive evaluations using an overall sensing ability score based on the SPOTIS method demonstrate that OCL consistently achieves superior performance across all path loss conditions, confirming its effectiveness and robustness for dynamic NOMA spectrum sensing.

## Figures and Tables

**Table 1:** Classification performance of OCL against baseline LightGBM.

Class	Baseline LightGBM			OCL Method		
	Precision	Recall	F1 Score	Precision	Recall	F1 Score
$\alpha_{1}=0.2$ and $\alpha_{2}=0.8$						
0	0.7140	0.9167	0.7736	0.6519	0.9739	0.7811
1	0.7119	0.6248	0.6791	0.7884	0.6340	0.7028
2	0.6622	0.3577	0.4372	0.7228	0.3604	0.4810
3	0.5655	0.7632	0.7008	0.6469	0.7998	0.7153
$\alpha_{1}=0.4$ and $\alpha_{2}=0.6$						
0	0.6991	0.9331	0.8090	0.6926	0.9917	0.8156
1	0.7437	0.4614	0.5599	0.8126	0.5450	0.6524
2	0.5621	0.4617	0.5440	0.8982	0.4192	0.5716
3	0.6479	0.7737	0.6543	0.5875	0.8574	0.6972

**Table 2:** Comparison of $P_{d}$ for NOMA-Based Spectrum Sensing Methods.

d	$\alpha_{1}=0.2$ $\;\mathrm{and}\;\alpha_{2}=0.8$					$\alpha_{1}=0.4$ $\;\mathrm{and}\;\alpha_{2}=0.6$				
	EBSS-RF	FBSS-LR	FBSS-OCL	1DCNN	OCL	EBSS-RF	FBSS-LR	FBSS-OCL	1DCNN	OCL
10	1	0.958	0.968	1	1	1	0.846	0.853	1	1
20	1	0.331	0.518	1	1	1	0.531	0.623	1	1
30	0.956	0.401	0.273	0.999	0.961	0.977	0.481	0.412	1	0.976
40	0.854	0.358	0.244	0.970	0.860	0.973	0.395	0.284	0.975	0.974
50	0.689	0.282	0.147	0.893	0.633	0.783	0.308	0.150	0.813	0.792
60	0.627	0.220	0.085	0.776	0.624	0.453	0.229	0.080	0.618	0.391
70	0.511	0.186	0.053	0.632	0.480	0.205	0.199	0.046	0.466	0.339
80	0.491	0.162	0.039	0.503	0.340	0.268	0.168	0.030	0.387	0.313
90	0.406	0.142	0.029	0.411	0.416	0.381	0.151	0.022	0.336	0.382
100	0.512	0.141	0.023	0.338	0.443	0.513	0.144	0.021	0.298	0.456
110	0.469	0.135	0.022	0.293	0.436	0.513	0.132	0.016	0.272	0.461
120	0.357	0.131	0.020	0.256	0.344	0.392	0.123	0.0156	0.259	0.377
130	0.243	0.124	0.018	0.239	0.238	0.259	0.120	0.0150	0.245	0.255
140	0.172	0.124	0.017	0.229	0.166	0.181	0.121	0.0142	0.237	0.169
150	0.130	0.121	0.017	0.214	0.115	0.128	0.117	0.0136	0.234	0.113

**Table 3:** Comparison of $P_{fa}$ for NOMA-Based Spectrum Sensing Methods.

d	$\alpha_{1}=0.2$ $\;\mathrm{and}\;\alpha_{2}=0.8$					$\alpha_{1}=0.4$ $\;\mathrm{and}\;\alpha_{2}=0.6$				
	EBSS-RF	FBSS-LR	FBSS-OCL	1DCNN	OCL	EBSS-RF	FBSS-LR	FBSS-OCL	1DCNN	OCL
10	0.115	0.337	0.042	0.447	0.056	0.088	0.332	0.037	0.541	0.046
20	0.113	0.344	0.040	0.443	0.054	0.078	0.321	0.036	0.546	0.046
30	0.110	0.340	0.038	0.443	0.054	0.079	0.332	0.033	0.559	0.046
40	0.104	0.342	0.042	0.452	0.057	0.085	0.343	0.032	0.548	0.044
50	0.112	0.341	0.044	0.446	0.059	0.087	0.329	0.034	0.575	0.043
60	0.106	0.339	0.044	0.441	0.053	0.085	0.334	0.030	0.588	0.041
70	0.105	0.348	0.043	0.446	0.055	0.082	0.334	0.036	0.446	0.044
80	0.107	0.349	0.039	0.441	0.050	0.083	0.318	0.032	0.553	0.042
90	0.109	0.342	0.035	0.441	0.055	0.085	0.349	0.038	0.557	0.045
100	0.114	0.345	0.040	0.433	0.056	0.082	0.332	0.038	0.556	0.045
110	0.116	0.341	0.039	0.448	0.053	0.084	0.339	0.031	0.552	0.047
120	0.118	0.343	0.042	0.443	0.053	0.081	0.337	0.034	0.554	0.047
130	0.111	0.352	0.042	0.438	0.051	0.080	0.346	0.033	0.552	0.047
140	0.113	0.345	0.046	0.441	0.057	0.089	0.329	0.033	0.560	0.048
150	0.110	0.341	0.040	0.441	0.051	0.086	0.322	0.032	0.553	0.044

## Data Availability

The data presented in this study are available on request from the corresponding author.

## Abbreviations

The following abbreviations are used in this manuscript:

OCL	Objective-driven cost-sensitive learning
NOMA	Nonorthogonal multiple access
CR	Cognitive radio
PU	Primary user
SU	Secondary user
FBSS	Feature-based spectrum sensing
EBSS	Eigen-based spectrum sensing
FBSS-LR	FBSS with logistic regression
FBSS-OCL	FBSS with cyclostationary features
